# Intramural tracheal bronchogenic cyst: a case report

**DOI:** 10.1186/2193-1801-3-262

**Published:** 2014-05-23

**Authors:** Go Ohba, Miki Toma, Koji Komori, Seiichi Hirobe, Ryuji Fukuzawa

**Affiliations:** Department of Surgery, Tokyo Metropolitan Children’s Medical Center, Tokyo, Japan; Department of Pathology, Tokyo Metropolitan Children’s Medical Center, Tokyo, Japan

**Keywords:** Bronchogenic cyst, Intramural, End-to-end anastomosis

## Abstract

Intramural bronchogenic cysts are extremely rare. We describe the case of an intramural bronchogenic cyst in a 2 year old boy who underwent tracheal resection and end-to-end anastomosis.

## Case report

**Figure 1:**
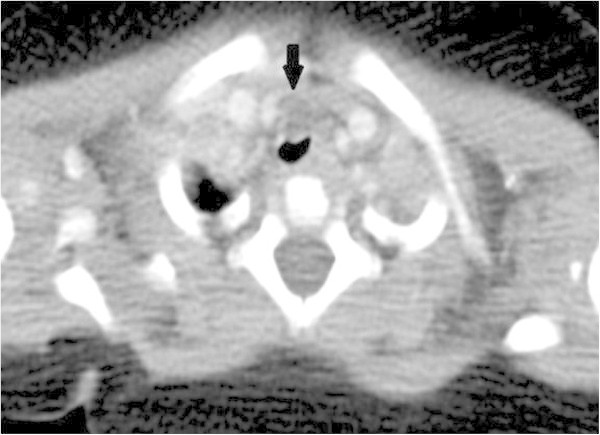
**CT findings: a cystic mass compressing the anterior wall of the trachea (Arrow).**

A 2 year old boy had suffered from inspiratory and expiratory wheezing since infancy, which was refractory to medications. Further examination with computed tomography (CT) revealed an unenhanced tracheal mass compressing the trachea on the left (Figure 1).

**Figure 2:**
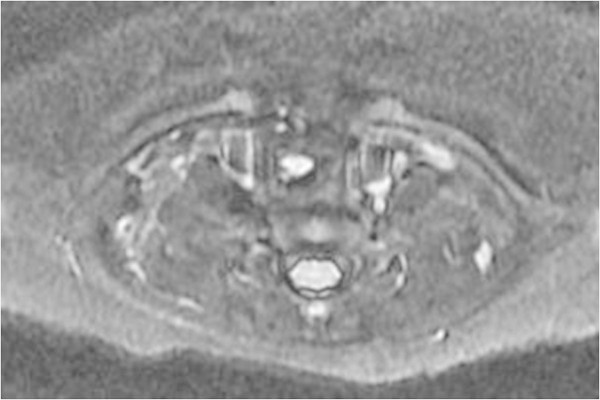
**The mass was of high intensity in T2-weighted MRI images.**

**Figure 3:**
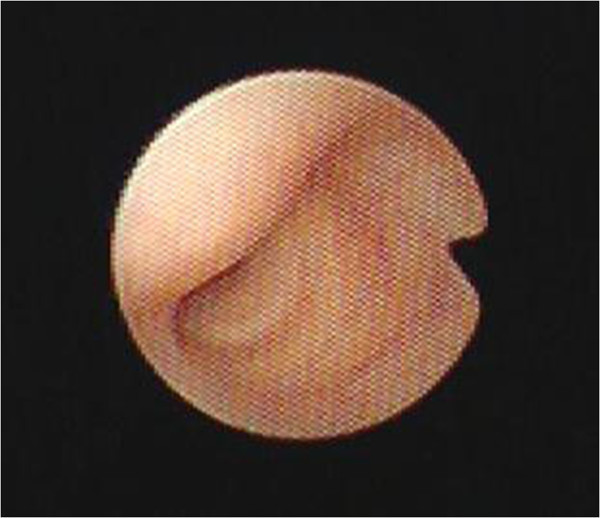
**Bronchoscopy shows a smooth mass, causing stenosis of the trachea.**

He was transferred to our institution for definitive treatment. On admission, chest radiography revealed apparent segmental narrowing of the mid-trachea, suggesting the existence of a paratracheal mass. The mass was of high intensity in the T2-weighted and isotense in the T1-weighted images in magnetic resonance imaging (MRI) (Figure 2). Flexible bronchoscopy was performed indicating a smooth protrusion of antero-lateral wall of the trachea, and the tracheal lumen was severely occluded (Figure 3). These results suggested a probable diagnosis of bronchogenic cyst arising from outside the tracheal wall and compressing the tracheal lumen.

**Figure 4:**
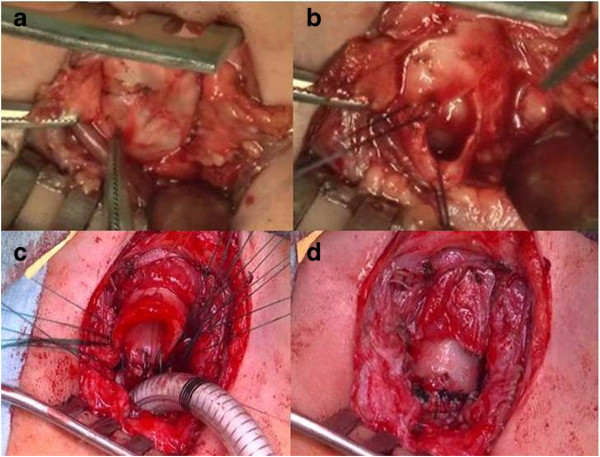
**Interpretive findings (ab: first operation cd: second operation). a**: The tracheal wall was bulging so that the mass was thought to be present intramurally. **b**: Mucus leaked when the wall was pinched and tracheal mucosa was exposed. **c**: Tracheal resection and end-to-end anastomosis was performed under operative field intubation. **d**: After end-to-end anastomosis.

**Figure 5:**
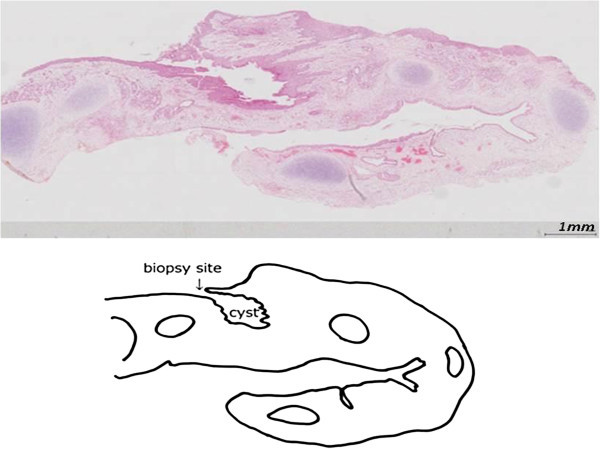
**The cyst wall was lined by respiratory epithelia and the wall contained cartilage.**

The operation was performed through a cervical transverse incision. The trachea was exposed and no mass was found in the paratracheal area. The anterior wall of mid-trachea was found to be bulging as the mass was within the tracheal wall. The bulging wall was thin, so that the contents could be seen through the wall. Mucous fluid spilled out when the wall was pinched. Because a bronchogenic cyst arising from the tracheal wall had not been expected, the possibility of malignant tumor could not be ruled out. A biopsy of the wall followed by definitive surgery was planned. Pathologically, the contents of the wall were dense fibrous tissue, epithelial tissues and cartilaginous tissue. This result supported the diagnosis of an *intramural bronchogenic cyst*. Definitive surgery was performed a week after the previous operation. The affected tracheal segment was completely resected and an end-to-end anastomosis was performed to restore the trachea (Figure 4). The resected specimen proved to be a cystic lesion within the tracheal wall indicating an intramural bronchogenic cyst. The cyst wall was lined by respiratory epithelia and the wall contained cartilage (Figure 5). It was diagnosed as an intramural bronchogenic cyst.

The postoperative course was good and there is no recurrence one year after surgery.

## Discussion

Bronchogenic cysts develop from abnormal budding of the tracheal diverticula during the embryological period. The exact time of these events has not been ascertained. Cysts are lined by epithelium, and their wall often contains mucous glands and cartilage.

Bronchogenic cysts can be classified into three categories in relation to the trachea as *paratracheally*, *intraluminally*, and *intramurally*. The localization of cysts between the mucosa and submucosa layer is called *intraluminal* and between the cartilages and adventitia is called *intramural*. In fact, the differentiation is complicated without pathological findings and cannot be distinguished before the operation (Stewart et al. 2002). Paratracheal cysts are found in various regions, depending on the level at which the abnormal budding occurred in the development of the tracheobronchial tree. Most cysts are found along the tracheobronchial tree, but have been reported to occur in various regions, such as neck, tongue, esophagus, heart, retroperitoneum, and mesentery (Sauvat et al. 2006; Antoniou et al. 2012; Govaerts et al. 2012; Petrina et al. 2010).

Making preoperative diagnosis of bronchogenic cysts can be difficult. Chest radiographs are ineffective for accurate preoperative diagnosis, but the diagnosis can be made with 69.2% and 100% accuracy in CT scans and MRIs respectively (Kanemitsu et al. 1999). MRI proved very useful for making diagnosis, but there are some reports describing that the diagnosis of bronchogenic cysts was difficult even with MRI (Sauvat et al. 2006). Transtracheal endoscopic ultrasonography is reported to be useful in adults (Anantham et al. 2011), however, it has not been reported in children.

Intramural bronchogenic cysts are extremely rare and only two reports were found (Wenig and Abramson 1987; Klin et al. 1994). These reports also could not be diagnosed as intramural lesions prior to surgery. Diagnosis was usually performed by CT or MRI. The preoperative diagnosis was bronchogenic cyst in our case, and it was expected to be located beneath the anterior cervical muscles attaching to the tracheal wall. However, the trachea was exposed and the anterior wall of trachea was found to contain the cystic mass. The cyst was not expected to be intramural therefore the decision was made not to perform definitive surgery. The pathological diagnosis of bronchogenic cyst supported our plan for complete resection of the affected trachea.

In this case, as the patient had developed respiratory symptoms since infancy, the surgical procedure was necessary. Treatment of asymptomatic bronchogenic cysts is controversial. But carcinoma arising from a bronchogenic cyst has been reported in an 8-year-old girl (Suen et al. 1993). Other symptoms, such as infection, bleeding and respiratory distress can develop, so the need for surgical excision in order to avoid those complications can be emphasized.

There was discussion as to whether complete resection of the affected tracheal rings was necessary or excision of the cyst followed by patch closure of the defect of tracheal wall would be sufficient. Complete resection was chosen because the length of the affected tracheal rings was less than 2 cm, end-to-end anastomosis of the trachea would be relatively safe without tension. Also, tracheomalacia might develop due to the defect of the anterior wall of the trachea in patch closure.

Excision of the cyst is apt to be conservative and complete excision is recommended. Recurrence 25 years after incomplete resection has been reported (Read et al. 1991). Although paratracheal cysts can be ablated from the tracheal wall, intramural cysts require tracheal wall resection for complete resection. Thoracoscopic laser excision or fenestration is sometimes useful, but the tracheal aventitia was too thin in this case therefore there was the risk of perforation. Drainage was also reported but long-term follow up is indicated to detect recurrence (Read et al. 1991; Li et al. 2010). Recently, CT-guided percutaneous treatment of bronchogenic cysts with needle aspiration and sclerotherapy was reported, but this technique was a small case series and further investigation is necessary (Li et al. 2010). As a whole, we considered tracheal resection as safe and assured complete resection.

## Summary

Intramural bronchogenic cysts are extremely rare and preoperative diagnosis is difficult. Intramural bronchogenic cysts should be considered when cystic tumors are close to the trachea, with tracheal resection and end-to-end anastomosis a safe surgical procedure for complete resection.
